# Meteorological Register

**Published:** 1814-05

**Authors:** C. Blunt

**Affiliations:** Philosophical Instrument Maker, No. 38, Tavistock-Street, Covent-Garden


### METEOROLOGICAL REGISTER.

*From March 25th, to April 25th,* 1814.

Kept by C. BLUNT, Philosophical Instrument Maker, No. 38, Tavistock-Street, Covent-Garden.

| Moon. | Day. | Wind. | Barometrical Pressure. Max. | Min. | Temperature. Max. | Min. | |
|---|---|---|---|---|---|---|---|
| | 26 | W | 29·79 | 29·63 | 51 | 44 | Fair |
| | 27 | W | 29·88 | 29·84 | 57 | 38 | Fair |
| ☽ | 28 | SW | 29·84 | 29·60 | 60 | 42 | Fair |
| | 29 | SE | 29·70 | 29·52 | 59 | 37 | Cloudy |
| | 30 | W & SW | 29·91 | 29·77 | 60 | 38 | Fair |
| | 31 | W | 29·90 | 29·81 | 50 | 47 | Cly&Rain |
| | 1 | W | 29·70 | 29 52 | 51 | 38 | Cloudy |
| | 2 | W | 29·50 | 29·47 | 52 | 45 | Rain |
| | 3 | SW | 29·60 | 29·56 | 59 | 38 | Fair |
| ○ | 4 | ENEtoSE | 29 69 | 29.61 | 58 | 35 | Fair |
| | 5 | E by S | 29·86 | 29·73 | 57 | 36 | Fair |
| | 6 | S by W | 30·07 | 29·93 | 58 | 38 | Fair |
| | 7 | SE | 30.19 | 30·02 | 56 | 39 | Fair |
| | 8 | SSE | 30·22 | 30·18 | 56 | 34 | Fair |
| | 9 | E by S | 30·20 | 30·10 | 57 | 37 | Fair |
| | 10 | E by S | 30·10 | 30·01 | 62 | 38 | Fair |
| | 11 | E by S | 30·— | 29·93 | 64 | 39 | Fair |
| ☾ | 12 | SE | 29·86 | 29·82 | 68 | 48 | Fair |
| | 13 | SE | 29 83 | 29.70 | 73 | 44 | Fair |
| | 14 | S | 29 65 | 29·54 | 70 | 44 | Fair |
| | 15 | S by W | 29 56 | 29·49 | 70 | 47 | Rain |
| | 16 | W | 29·54 | 29·49 | 61 | 48 | Fair |
| | 17 | SW | 29·61 | 29 56 | 60 | 47 | Fair |
| | 18 | SW | 29·62 | 29·54 | 69 | 48 | Fair |
| | 19 | SW | 29·71 | 29·66 | 69 | 46 | Fair |
| ● | 20 | SW | 29 71 | 29·68 | 68 | 46 | Fair |
| | 21 | W by S& NW | 29·75 | 29·70 | 59 | 39 | Cl.& Showers |
| | 22 | NW | 30·— | 29·80 | 59 | 38 | Cl.& Showers |
| | 23 | NW | 30·02 | 29·86 | 58 | 39 | Cl.& Showers |
| | 24 | NW | 29·84 | 29 80 | 57 | 40 | Brisk Wind & Showers |
| | 25 | NE | 29·84 | 29·84 | 50 | 39 | Cl.& Showers |

RESULTS.

| | | | | | |
|---|---|---|---|---|---|
| Mean barometrical pressure | 29·71 | | Mean temperature | | 50·78 deg. |
| Maximum 30·22 inc. | wind at | SSE | Maximum 73, | the wind at | SE |
| Minimum 29·47 | ——— | W | Minimum 34, | ——— | SSE |

*Scale exhibiting the prevailing Winds during the Month.*

| N | NE | E | SE | S | SW | W | NW |
|---|---|---|---|---|---|---|---|
| 0 | 1 | 0 | 10 | 1 | 9 | 6 | 4 |

| | Mean barometrical pressure. | Mean temperature. |
|---|---|---|
| From the first quarter on the 28th March, to the full moon on the 4th April | 29·67 | 48·3 |
| ——— full moon, to the last quarter on the 12th | 29·96 | 47·0 |
| ——— last quarter, to the new moon on the 20th | 29·50 | 55·3 |
| ——— new moon, to the first quarter on the 26th | 29·70 | 52·5 |

April 26th.—Few remarks are necessary to assist the preceding observations.

vations. After the sudden breaking up of the frost last month, the weather became gradually more mild, and the temperature progressively increased until the 21st of the present month. During this period the wind has been variable between the limits of the quarters S and W. The rapid progress of vegetation was distinctly visible from day to day. In a single week (from the 11th to the 18th) nature assumed her accustomed verdancies. This had hitherto been checked by what we may consider the encroachment of winter, and is again materially impeded, for since the 21st the wind has been shifting towards the north, the days are checquered with frequent showers and partial gleams of sunshine, and the atmosphere is consequently unusually damp and chilly, although the general temperature of the twenty-four hours is not much decreased.

Catarrhal and pulmonic affections still claim the chief attention of the practitioner. Hooping cough has become more frequent. Several cases of ophthalmia with febrile symptoms have occurred; and anasarca, chiefly of the lower extremities, has much prevailed, especially in females, who did not appear to have lived intemperately.

